# Teaching Mindfulness-Based Programs online: perceived costs and benefits

**DOI:** 10.1186/s40359-025-02983-8

**Published:** 2025-12-09

**Authors:** Alison M. Burton, Rebecca S. Crane, Gemma M. Griffith

**Affiliations:** Centre for Mindfulness Research and Practice, School of Psychology and Sports Science, Bangor University, Bangor, Wales UK

**Keywords:** Mindfulness, Mindfulness-Based Programs (MBPs), Online, MBI:TAC, Mental health, Teachers, Qualitative

## Abstract

**Objectives:**

This study examines the demographics of those who teach Mindfulness-Based Programs (MBPs) online, and their experiences of the online teaching process. Understanding these issues could inform strategies to widen access and enables understanding of the challenges and opportunities of teaching MBPs online versus in-person.

**Methods:**

Data from 109 mindfulness MBP teachers were collected via a 50-question online survey, originating in the United Kingdom (UK). Responses were analysed using SPSS. Content Analysis was undertaken on the qualitative responses to identify key themes.

**Results:**

Teachers articulated some challenges in teaching MBPs online such as embodying mindfulness and safeguarding participants. Teachers were mostly positive about online teaching for reasons such as wider accessibility. The survey also highlighted the lack of demographic diversity amongst the online mindfulness teaching community.

**Conclusions:**

The field would benefit from further considering best practice concerning nuanced aspects of online MBP delivery, particularly around how embodiment is communicated and safeguarding participants. The challenges and opportunities identified in this study are likely to be applicable to other online learning and therapeutic environments. The apparent lack of diversity amongst MBP teachers could limit the potential for MBPs to impact broad sectors of societies, though it is unknown how representative the current sample is.

Over the last four decades, many people have reported experiencing significant health benefits from participating in Mindfulness-Based Programs (MBPs). MBPs draw from contemplative traditions and medicine, psychology, and education. They consider the causes of human suffering and routes to relieving it, training the participant in mindfulness meditation practice, and incorporating an experiential inquiry-based learning process which enables the participant to cultivate a changed relationship with their experiences [1]. MBPs have become extensively available and have been adapted from the original focus on stress reduction [2] and depressive relapse prevention [3] to meet the needs of those experiencing a variety of physical and psychological health and ongoing life challenges [4]. In the UK, the National Institute for Health and Care Excellence (NICE) recommend group mindfulness and meditation as a first-line treatment for less severe depression (defined as ‘traditional categories of subthreshold symptoms and mild depression’), and Mindfulness-Based Cognitive Therapy (MBCT) as a treatment option for preventing depressive relapse [5]. There is an ever-expanding list of recognised MBPs, from those designed to manage stress and depression and to cultivate compassion, to more specialised programs supporting people living with specific health challenges.

Mindfulness-Based Programs are defined by the British Association of Mindfulness-Based Approaches (BAMBA), [4] as being:

‘*Informed by a clear rationale; Teacher-led; Have been developed to be scalable; Have a set curriculum, typically at least eight sessions with 30 – 45 mins daily home practice, incremental development and experiential learning; and have a clear commitment to be evidence-based’.*

Zhang et al. [6] stated that MBPs improve many biopsychosocial conditions, including depression, anxiety, stress, insomnia, addiction, psychosis, pain, hypertension, weight control and cancer-related symptoms. The positive impacts identified include enhanced wellbeing and prosocial behaviours. They found MBPs to be supportive in a wide range of populations and contexts, including healthcare settings, schools and in the workplace, though further research is needed to develop evidence of their effectiveness for issues such as Post Traumatic Stress Disorder and eating disorders [6].

The advent of the COVID-19 pandemic in early 2020 and associated stresses meant that MBP participation seemed particularly relevant to many individuals who found themselves facing the challenges of adapting to the constraints and threats presented by a global pandemic. Large-scale lockdowns necessitated a rapid shift from in-person to online delivery. Prior to the pandemic, online delivery of MBPs was not considered mainstream, though there was emerging literature about its feasibility and effectiveness. Participants’ Perceived Stress Scale [7] scores significantly improved after completing an online MBP, and results were comparable with those achieved in-person [8]. A meta-analysis of the effect of online MBPs on mental health and wellbeing found that guided online MBPs had a small but significant beneficial effect on depression, anxiety, and stress, with a moderate effect size [9]. MBPs could be a convenient and cost-effective strategy compared with traditional in-person interventions [10], particularly for busy, marginalised populations with access to digital platforms.

The lockdowns and pivot to online MBP teaching vastly increased opportunities for mindfulness participants to attend an MBP from home: geographical obstacles disappeared, and financial barriers were mitigated. Indeed, many MBP teachers offered reduced cost or even free online MBP courses during this period. Less positively, there were anecdotal concerns expressed within the mindfulness field about the quality of some online MBP delivery, lack of pre-course participant assessment, and consequent safeguarding worries regarding potentially vulnerable participants.

In 2020 it seemed that the pandemic might catapult MBP delivery further into the mainstream as the shift from in-person to online delivery seemed to offer huge scope to broaden access to MBPs. Mindfulness teacher-training organisations, recognising the potential challenges of online training to the integrity of mindfulness teaching, promptly published Good Practice Guidance for online MBP delivery. These guidelines suggested adaptations for teaching MBPs in an online setting to help mitigate some of the new challenges identified [11]. Dobkin wrote to the editor of *Mindfulness* outlining her experiences of teaching online [12] and citing that the initial concerns identified when contemplating the shift to online delivery had been replaced by a recognition that the modality is effective, despite not being able to connect with participants in the same room. Further research has been undertaken about the efficacy of MBPs online, and this has largely supported the feasibility and acceptability of online MPBs. Since the pandemic, Sommers-Spijkerman et al. have updated their 2016 meta-analysis of Randomised Control Trials [13] and reported that online MBPs were booming and seemed beneficial for improving mental health outcomes in a broad range of populations. Yeun and Kim conducted a systematic review of Randomised Control Trials [14], exploring the psychological effects of online-based MBPs during the COVID-19 pandemic. They found that MBPs offered effective complementary interventions for clinical populations, healthy individuals, and healthcare workers experiencing psychological challenges. A study investigating efficacy of mindfulness training for parents of children suffering from a health condition found that improvements in wellbeing and self-compassion were comparable between groups that were run in-person and online [15]. A systematic review into feasibility, acceptability, safety, and efficacy of delivering MBPs via video conferencing determined that the online modality was effective, though did not draw firm conclusions about feasibility, acceptability and safety since few studies had explored these aspects [16]. Therefore, there is increasing evidence to support the efficacy for participants of online MBPs, although a dearth of data regarding MBP teachers’ experiences of acceptability and safety when delivering online.

Research into therapists’ experiences of delivering online (non-mindfulness) therapy may be helpful when considering online MBPs. Therapists’ experiences of working online were more positive than expected, but challenges were identified [17]. The most significant of these was limited physical contact and body language (both of which could be further diminished by technological disruptions) which had a direct impact on developing the therapeutic relationship. Other observations included difficulties related to combining the home environment with the therapeutic space, for example more interruptions from family members or technological devices, and client behaviours that would not happen in an in-person session (e.g., the presence of children in the therapy setting, and drinking alcohol during therapy). Therapists reported that online therapy was not suitable for more complex psychopathology or mental health issues, such as trauma or personality disorders. They noted their lack of training and guidance in offering online therapy and considered this modality to be more subtle and complex than in-person therapy. Whilst MBPs are not therapies, they share many common characteristics, and it seems likely that similar challenges (for example concerning relationship building and practicalities like interruptions) would be experienced by online MBP teachers.

Because of the complex processes at play during an MBP, it also seems possible that there may be pedagogical implications for teachers when facilitating an MBP-training experience online. MBP group processes typically follow a characteristic path: teachers build and steer the group “vessel” in a way that builds a specific culture and sense of safety, where the group is facilitated to encourage the sharing of communal experiences that enhance learning [18]. The importance of a rich group learning environment in MBP contexts has been highlighted, with research suggesting that the experience of being in a group is potentially critical to participant outcomes [19]. The importance of teacher and participants together sustaining an atmosphere of mindfulness and in so doing, co-creating a safe and ethical space in which they facilitate transformation of each other has also been identified [20]. A study into online group therapy [21] found that whilst it can be effective, it is important to adapt to the online environment, to manage the setting and boundary issues and to find ways of compensating for the lack of physical presence, suggesting that explicit training for the online environment would be beneficial. It seems possible that delivering MBPs to participant groups who never meet in-person may disrupt this important facet of the MBP experience. We therefore need to understand whether teachers perceive group process to be impacted when delivering MBPs online.

An exploration of the frequency of adverse and harmful events for MBP participants (irrespective of delivery mode), concluded that although harm is probably rare, difficulties are seen as learning opportunities and that teacher skill is essential to managing those risks [22]. Safeguarding participants online may necessarily differ substantially from working with any participant difficulties and subsequent safeguarding during traditional in-person courses, and in the absence of research in this domain, teachers’ experiences of safeguarding online warrant exploration. As referenced above, it has been concluded that online therapy is unsuitable for severe psychopathology or mental health distress. This is because attendees require more contact and containment than is feasible online, and because micro-emotions cannot always be picked up via video [17], suggesting that participant safety during online MBPs is an area deserving of attention.

Given that that online MBPs are here to stay in the post-pandemic world and that research into online mindfulness, both pre- and post-pandemic, has largely focused on program efficacy, it seems vital to explore teachers’ views and experiences of delivering MBPs online to help to both optimise their potential and to mitigate any risks involved. Further, there is a lack of data regarding who teaches MBPs online. There is also no data regarding the number of trained or currently active mindfulness teachers either nationally or internationally. One of the aims of this study is therefore to learn something about the demographics of online mindfulness teachers, as we could find no prior studies investigating the demographics of MBP teachers in general. Understanding who is teaching MBPs online seems important if the mindfulness field is to develop ways to widen access to the full demographic of society. Critiques of the mindfulness field include awareness that the benefits of MBP training have been available to a relatively narrow demographic – white, middle class, and often female [23]. It is important to know more about who is teaching mindfulness online because if teachers have a very different background from that of potential mindfulness participants, individuals who might experience significant benefits from the training may not become aware of an opportunity to attend an MBP, or may not perceive it as sufficiently appealing, safe, or inclusive. Research in the therapeutic field found that patients expressed a strong preference to work with therapists from the same ethnic background, though the impact of race on therapeutic outcomes was negligible [24]. It is also important to know more about the range of mindfulness curricula that are being taught online, how they are taught, and to whom, not only because of its potential to improve wellbeing, but because of the risks that enhanced remote access to MBPs may pose to marginalised people.

This study is exploratory and adopts an interpretivist approach, its aims including: 1) to gain a sense of the demographics of online MBP teachers; 2) to identify which MBPs are being taught online and to whom, and 3) to gain understanding of teachers’ views and experiences of teaching MBPs online, particularly regarding any difficulties encountered.

## Method

### Participants

A total of 109 MBP teachers were recruited. Inclusion criteria were: 1) over the age of 18 and of any gender and geographic location, and 2) had delivered at least one MBP fully online. We used the Crane et al. definition of an MBP to determine inclusion [1], considering responses from participants who had delivered teaching that maintained fidelity to what the researchers consider essential MBP components. There were no exclusion criteria regarding in-person teaching experience.

Mindfulness teaching is not an internationally regulated profession, and there is no obligation for mindfulness teachers to register with any association. To recruit participants, we therefore combined a social media outreach campaign with direct contact with international training organisations. The social media recruitment campaign was launched on Facebook and X (formerly Twitter) via the Centre for Mindfulness Research and Practice and first author’s social media accounts, and via ‘snowballing’ where we invited followers of those accounts to engage with the survey and to share survey information and links with their own mindfulness contacts. We also directly contacted international MBP teacher training organisations and associations of MBP teachers by email, asking them to share information about the study and the link to the online survey with their mailing lists and to distribute the advertising imagery and narrative we provided via their own social media accounts. The nature of the recruitment campaign means that it is not possible to assess the response rate because there is no organisation or source of data that can identify the complete population of active mindfulness teachers, and we do not know how many people engaged with the advertisements.

### Procedure

Ethical approval for this project was granted by Bangor University Ethics Committee in August 2021. Participants were recruited via social media and other online outreach forums which provided the study survey link. Participants read the information sheet and consented on the opening page of the study; they could not access the online study without first consenting. No incentive was offered for participating in the survey. The majority of questions were optional and could be omitted if the participant preferred not to answer. The survey opened on 8 February 2021 and closed on 30 April 2022.

The survey was designed by the research team who are also MBP trainers and teachers with experience of delivering MBPs online. Extensive consideration was given to core elements of mindfulness teaching that seemed to be in some way altered when programs are delivered online rather than in-person, for example teacher embodiment and participant safeguarding. A focus group for usability testing was established. The draft survey was sent to 18 experienced MBP teachers who were invited to feedback on the usability and content of the survey. Eight of these teachers then met with the authors to evaluate each survey question. The feedback was positive with no significant changes identified. The group did identify opportunities to improve question specificity to eliminate potential ambiguity. This typically involved changing an open-ended question to a multiple-choice format or providing an additional space for clarifying comments. There were no questions added or removed. The review process ensured that the survey addressed core themes and could also be completed readily within the anticipated timeframe. All proposed amendments were adopted in the survey and as they were a matter of slight re-wording for clarity, it was not necessary to submit the survey for an ethical re-review, in accordance with university ethics committee standards.

The survey was designed to be completed online wherever the participant was located and to take around 20–30 min to complete. It opened with 12 multiple-choice questions about the participants’ demographics (e.g., gender, age, ethnicity, health, residence, household income, highest level of education, religious beliefs). These were followed by 15 questions about their experience of teaching an online MBP, for example which MBPs they taught, and the populations recruited. These questions were a combination of quantitative multiple-choice and related qualitative open-ended questions, allowing teachers to supplement an answer with a comment. The next ten questions considered more practical online teaching experiences such as timescale and duration of sessions. Teachers were then asked to consider ten further questions relating to their interaction with course participants. Where possible the questions incorporated pedagogical aspects of online teaching experiences and were based on the six domains of the Mindfulness Based Interventions: Teaching Assessment Criteria (MBI:TAC) which is an MBP teaching assessment tool [25].

To obtain a sense of similarities and differences when delivering MBPs online versus in-person, teachers were asked to compare experiences of their most recent online course with their general experiences of in-person teaching (if they had taught in-person). Questions included overall satisfaction with the course, experiences of embodying mindfulness, participant engagement, teachers’ self-reported ability to convey course themes online, sense of connection with the group and the group process, extent of participant disclosures, and their self-reported ability to manage participant distress. The final three questions asked for reflections of experiences of teaching online: its advantages and disadvantages, and any other observations. A total of 24 qualitative questions was included in the survey. Of these, nine were standalone qualitative questions and the remainder offered the participant an opportunity to comment further on their initial response to the quantitative component of a question.

The risk of distress to survey participants was considered minimal as the questions focused on professional issues such as their experiences of teaching MBPs. For safeguarding purposes, the participants were given the researcher’s contact details and invited to contact them if they experienced distress having undertaken the survey. No participants contacted the researcher for this (or any other) reason.

### Data analysis

IBM SPSS (Version 27) was used, and the data set was cleaned and adjusted in the following ways: (i) one participant wrote that the fee charged for their MBP course was £3000 per person. The figure was excluded from the data due to the likelihood of this being a typing error; (ii) another participant wrote they had not taught any online courses – however, as the survey was designed for teachers who had taught online and their other responses indicated that they had taught online, we concluded this was likely a typographical error and included their data and removed their ‘zero’ response on that item. Where financial information was provided in other currencies, fees were converted to British Pounds (GBP) using an average of the rates in effect during the survey period.

Responses to the qualitative, open-ended questions were analysed using content analysis [26] to identify themes and patterns in the data. Qualitative content analysis aims to identify specific categories that are refined in an iterative feedback loop to enhance relevance and credibility [27]. The first author, who carried out the content analysis, is trained to teach mindfulness and had not taught an MBP online, reducing the risk of preconceptions about teachers’ experiences of having done so. They were reflexive in their approach to data analysis, carefully noting any internal responses to the data and actively cultivating mindful attitudes, particularly ‘beginner’s mind’, whilst analysing the data to help minimise risk of unconscious bias. Every response to a specific qualitative question was saved into a separate master spreadsheet for that question. An initial review of the content highlighted recurring themes for each question, so that an inductive coding scheme for that question could be developed, based on the data collected from the survey rather than on the first author’s expectations. Comments that were a strong fit for those themes were moved into the appropriate section of the tabulated document. As the process continued, additional sub-themes were generated, necessitating a review of the earlier classification and refinement of the coding scheme and analysis. For example, an overarching reason for participants having dropped out of an MBP was identified as lack of time, so all comments mentioning time were allocated to that theme. As this iterative process continued, further themes were identified, such as a) a general lack of time, b) a lack of time because of work commitments and c) a lack of time due to family obligations, enabling sub-classification within the broad theme of ‘time’. Having classified all comments that related to the dominant themes, it was then possible to identify commonalities amongst the remaining comments. Most comments were typically a sentence in length which rendered identification of a central theme relatively straightforward. Once the only comments that remained were off topic and therefore discarded, the process was complete for a particular question and was repeated for the next.

In some instances, a respondent made several points in their response to an open-ended qualitative question, making classification more challenging. These responses were therefore split into shorter phrases which were allocated into each applicable content category. For example, within the question about the diversity of participants, a respondent may have mentioned several demographic characteristics, such as ethnicity, age, and gender. This means that where a question shows *N* total responses, there could have been fewer teachers who responded to a particular question, as some participants offered multiple relevant observations relating to different themes within their single answer, so in effect gave more than one answer to some questions. This led to *N* exceeding the total number of survey respondents on occasion.

The third author, an experienced qualitative researcher, reviewed and triangulated the analysis on a sample basis, reviewing approximately one third of the data coding. The coding adopted by the first researcher was aligned with the codes the third author independently generated. There were no disagreements regarding categorisation of the themes, perhaps because the data had been collected via a standardised written survey and so answers were very short.

## Results

### Demographics of mindfulness-based program teachers

The majority of MBP teachers who responded to the survey were cis-gender females (81.1%), with a mean age of 52.95 years (*SD* = 9.74 years, *Range* = 27 to 73 years). Table 1 shows education level, employment status, household income, ethnicity, country of residence and religious affiliation. Most participants reported a very high level of education: 94.2% had graduated from university, and 74.0% had a postgraduate qualification. Regarding employment status, 45.5% were employed full-time and 39.4% part-time, with 8.1% describing themselves as unemployed. Of those who responded, 83.4% reported a total household income of more than £30,000 per annum, and 24.7% had a household income over £70,000 per year. In 2021 the UK median household disposable income was £31,400 [28] suggesting that the majority of MBP teachers’ household were earning around the median or above the national average in the UK. Most teachers described themselves as white (80.2%) and a further 5.9% identified as Asian, whilst 11.9% of participants did not specify their ethnicity. Participants were resident across 19 countries. Of these, 88.6% were based in Europe, and the majority of these (61.5%) lived in the UK, though responses were also received from teachers living in United States of America (USA), Canada, Australia, Malaysia, and China. Of the 88 teachers who responded to the question about religious belief, around 28.7% considered themselves to be atheist or agnostic, 25.3% reported they were Buddhist or had Buddhist leanings, and 21.8% described themselves as Christian. See Table 1 for further details of demographics.

**Table 1:** Participant demographics

	*N:*	104	
**Highest Academic Qualification**	*n*	%	
Non-graduate	7	6.7	
First degree*	21	20.2	
Postgraduate degree*	77	74.0	
**Employment Status**	*N:*	99	
	*n*	%	
Employed full-time	45	45.5	
Employed part-time	39	39.4	
Retired	6	6.1	
Student	1	1.0	
Unemployed	8	8.1	
	*N:*	93	
**Household Income (GBP)**	*n*	%	
Under £20,000	14	15.1	
£20,000-£29,999	9	9.7	
£30,000-£49,999	25	26.9	
£50,000-£69,999	22	23.7	
> £70,000	23	24.7	
	*N:*	101	
**Ethnicity**	*n*	%	
Asian	6	5.9	
Latino	1	1.0	
Mixed race	1	1.0	
White	81	80.2	
Unspecified	12	11.9	
	*N:*	105	
**Residence**	*n*	%	
Australasia	1	1.0	
Asia	2	1.9	
Europe +	93	88.6	
N America	9	8.6	
Religion	*N:*	87	
	*n*	%	
Atheist/Agnostic	25	28.7	
Buddhist/secular	22	25.3	
Christian	19	21.8	
Humanist	1	1.1	
Spiritual	9	10.3	
Jewish	1	1.1	
Muslim	2	2.3	
Quaker	1	1.1	
Unsure/Other	7	8.0	
		+ Of which UK: *n* = 67 (63.8%)	

*or other equivalent qualification

### Teaching MBPs online: curriculums, fees, and populations

Table 2 shows the MBP-related teaching activities of teachers, the MBP curriculum they most recently taught online, and the populations taught. There was a wide variation in both online and in-person teaching experience, although most teachers had delivered multiple MBP courses online (*M* = 7.15, *SD* = 7.55, *Range* = 1—50) as well as in-person (*M* = 12.9, *SD* = 14.41, *Range:* 0—90). Teachers’ most recent online course had typically been delivered within the last month (49.5%) with 34.7% having taught online within the last six months. Lower numbers of teachers had recently taught in-person MBP courses: 25.2% had taught in-person within the last month but a majority (44.9%) had not taught an in-person MBP within the last year. In addition, 11.2% had never delivered an in-person course (and were thus unable to answer the questions comparing their online experiences to in-person teaching). Teachers were typically experienced—only 6.4% of teachers stated that they did not engage in any other professional mindfulness activities in addition to teaching MBPs (such as training, supervision of mindfulness teachers, leading retreats, or academic study).

**Table 2:** Participant teaching

	*N:*	109
**Other Mindfulness Activities**	*n*	%
Teacher Trainer	39	35.8
Supervisor	32	29.4
Researcher	31	28.4
Operations	27	24.8
Therapist	26	23.9
Retreat lead	25	22.9
Other	9	8.3
None	7	6.4
	*N:*	99
Most Recent Online MBP*	*n*	%
MBSR	41	41.4
MBCT (D)	15	15.2
MBCT-L	9	9.1
Compassion	9	9.1
Adaptation	9	9.1
MBChP	6	6.1
Health	5	5.0
FPFW	3	3.0
Advanced	2	2.0
	*N:*	109
**Population Taught**	*n*	%
Public	79	72.4
Other	35	32.1
Clinical	32	29.4
Schoolteachers	15	13.8
Schoolchildren	10	9.2
Prison	1	0.9

*or other equivalent qualification

Teachers charged a mean fee of £146.66 per person for their most recent online MBP (*SD* = £147.95, *Range* = £0—£700), though included in this average are 36% of teachers who reported that they offered their courses free of charge. When those free courses were excluded from the data, the mean fee was £232.04.

### Teachers’ views and experiences of online MBP teaching

How teachers orientate participants to the course is an important part of MBP teaching. It familiarises potential participants with what an MBP involves, gives them a flavour of the group setting, elicits a commitment to engage in active participation in the program, and is a safeguard to ensure that an MBP course is currently suitable for the participant [29, 30]. Around half of the teachers held a pre-course orientation meeting with the whole group, and a similar number spoke to participants on a one-to-one basis. A minority (6.4%) reported that they had not collected any pre-course information from participants.

In terms of teacher expectations of participant engagement online, 54.9% of teachers responded that they expected their participants to leave cameras on for the duration of the session. Of those teachers, 17.6% reported that they varied this requirement depending on the specific practice being delivered, whilst 26.5% allowed the participants to decide what suited them. The majority (81.2%) of MBP sessions lasted between 2 and 2 ¾ hours, the mean session time being 125.4 min (*SD* = 24.3, *Range* = 20 – 180 min). Almost all online MBP teachers reported that they felt either extremely (59.4%) or moderately (38.7%) technologically competent.

Table 3 summarises class size, teacher perceptions of group diversity, the frequency with which teachers undertook supervision and the nature of any technological challenges observed, including those experienced by course attendees. MBP teachers are encouraged to arrange regular supervision with an experienced mindfulness teacher to reflect on and inquire into personal processes and to receive feedback on teaching [3]. A monthly supervision cycle was most common (37.8%) though notably, 12.2% of teachers stated they did not engage in any mindfulness supervision. Undertaking regular supervision with an experienced MBP teacher is a UK Good Practice Guideline that must be adhered to if a teacher intends to seek listing with BAMBA [3].

**Table 3:** Online teaching experiences

**Number of Course Participants**	*N:*	100		
	*n*	%		
1	3	3.0		
2–4	19	19.0		
5–9	37	37.0		
10–14	22	22.0		
15–19	10	10.0		
20–24	6	6.0		
> 25	3	3.0		
**Diversity Online versus In-person **	*N:*	89		
	*n*	%		
Significantly more	23	25.9		
Somewhat more	19	21.3		
Similar	39	43.8		
Somewhat less	7	7.9		
Significantly less	1	1.1		
**Supervision Frequency**	*N:*	98		
	*n*	%		
Monthly	37	37.9		
Fortnightly	21	21.4		
None	12	12.2		
As required	11	11.2		
Weekly	11	11.2		
Occasional	6	6.1		
**Technological issues experienced**	Teachers' own		Participants'	
	*N:*	109	*N:*	109
	*n*	%	*n*	%
Connectivity	26	23.9	76	69.7
Breakout room issue	17	15.6	N/A	N/A
Distraction	12	11.0	N/A	N/A
Left people in wait room	6	5.5	N/A	N/A
Battery failure	1	0.9	15	13.8
Lighting	N/A	N/A	34	31.2
Microphone	N/A	N/A	50	45.9
Other	14	12.8	18	16.5

Teachers were provided with opportunities to expand on their experiences of teaching mindfulness online in a series of open-ended questions. The teachers who responded to questions about teaching experiences over the last five years had taught a mean of 5.81 courses in person and a mean of 2.68 courses online during that period and were therefore a relatively experienced group. Table 4 summarises the themes and subthemes identified.

**Table 4:** Content analysis online teaching experiences

**Diversity Online Compared with In-person**		
*N*: 77	*n*	
Geographical Location	32	*'Having people from China/Hong Kong/other European countries would not have happened when I was teaching face to face'*
Age	13	*'The age range of the participants was greater. On 1 course from 23 to 89’*
Ethnicity	7	*‘More diverse in terms of ethnicity’*
Health/Disability	6	*'I have had several participants on online courses who are housebound for physical/mental health reasons who would not have attended in-person'*
Education	3	*'Perhaps more people without college/university education*
Gender	3	*'Perhaps slightly more men than usual'*
Occupation	3	*'Online courses have permitted housewives to attend as well as employees attending during working hours'*
Income	2	*'There were people from low income, disability benefits, high income earners’*
No difference	2	*'Didn't impact on the nature/type of the participants'*
Miscellaneous	6	
**Teachers' Own Technological Issues**		
*N*: 13	*n*	
Platform	4	*'Zoom freezing PC upgrading zoom during session'*
Audio	2	*'People not hearing me well enough'*
Software	2	*'Moreover in PowerPoint I was unable to use the slideshow when sharing the screen'*
Competence	1	‘*Difficulties with sharing the screen and the participants being able to view what was being shared’*
Miscellaneous	4	
**Participants' Technological issues**		
*N*: 26	*n*	
Competence	7	*'unable to manage to log on to meeting or unmute meant thay (sic) could not join in'*
Hardware	4	*'Using very small screen to (sic) hard to see each other'*
Internet	4	*'Internet failure preventing attendance'*
Platform	3	*'inability of participant to log on due to incompatability (sic) of their device with MSTeams or CISCO'*
Video	3	*'couldn't use camera due to poor wifi'*
Audio	2	
Miscellaneous	3	
**Commitment to Home Practice**		
*N*: 37	*n*	
Similar Commitment	18	*‘When we have not been in lockdown, I haven't seen any difference between online and in person engagement’*
Less Commitment	6	*‘Participants missed the mutual support and catch ups you'd normally get [..]which I think helps them to feel part of something and therefore more likely to get stuck in*
Greater Commitment	3	*'Having sessions from home allowed some participants to see the possibility of integrating practices in daily life and contexts more easily and readily'*
Couldn't Tell	2	*'When the group is in-person, participants often bring their diaries and can be overheard talking about their meditation practice'*
Not Applicable	1	
Miscellaneous	7	
**Teachers' Experiences of Participants' Cameras being off**		
*N*: 87	*n*	
Negative Impact		
*- Discomfort*	20	*‘There were a few occasions when a participant consistently kept their camera off that I felt some distance’*
*- Group Process*	20	*'I noticed other participants interacted minimally with someone who had the camera off a lot of the time'*
*- Safety Concerns*	3	*'Feels safer to see all participants on the screen'*
No Impact/Permitted	17	*'Camera off participants have been in the minority really. I don't find that this has any significant impact on the experience as a teacher or an active participant'*
Not experienced	3	
Guidance Given	5	*'I indicate that temporary is fine, however, not for long periods as it’s not fair in those who are fully showing up’*
Follow up Contact	9	*'I had to phone people after the session to check they were OK*
Weave into Practice	5	*'I did not feel I could insist on cameras on, so I encouraged by invitational language.’*
Miscellaneous	5	
**Reasons for** **Participants Leaving the Program**		
*N*: 71	*n*	
Time Constraints		
*- Unspecified*	16	*'1 person dropped after class 3: felt overwhelmed with too many things going on personally at the same period’*
*- Work*	11	*'Work commitments made it difficult to keep up the practice'*
*- Family*	2	*'Mostly after about week 2/3. Most had emergency family issues- elderly ill parent or children at home'*
Change in Circumstance		
*- Family*	4	*'One drop out, Change in family circumstances. Week 5'*
*- Bereavement*	4	*'after week 5, death in the family'*
*- Illness*	4	*'one dropped out due to illness after week 2'*
*- Other*	3	
*- Relocation*	2	
Online Issues	8	*'Session 2—she dropped out because she fell asleep and found this to be particularly embarrassing online’*
Pre-existing Health Issue	4	*'One dropped out after week 3 due to increased anxiety'*
Dissatisfaction	4	*'session 3—MBCT not for them'*
No Reason Supplied	4	
Miscellaneous	5	*'Many small children appearing on screen, sitting on parents knees during the class. Participants being called away to deal with something*
		*Pets needing attention- I even opened my eyes during a guiding to find a woman with a python round her neck'*

When asked about perception of participant diversity online relative to an in-person course, eight sub-themes were identified (*n* = 77). These were: geographical diversity, participant age, ethnicity, health and disability, gender, education, occupation, and income. The most common response (*n* = 32) was that geographical diversity was increased. Some teachers (*n* = 13) noted that online courses appeared to attract more diverse age groups, specifically that there were more young people and a wider range of ages overall, though fewer older people joined online courses than in-person.

Fewer teachers (*n* = 13) responded to the open-ended question about technological difficulties. Of these, four had experienced issues with the online platform limiting access, freezing, or rendering it impossible to see all participants during their most recent online course. Slightly more teachers answered the question about their observations of participants’ technological difficulties (*n* = 26). The themes identified were competence and problems with internet, online platforms, video, and audio.

There were 37 responses to the question comparing participants’ commitment to home practice online versus in-person delivery. Of these, 18 reported that commitment to home practice seemed similar to their experiences during a typical in-person course. Three teachers thought that commitment was greater, six believed it was diminished online. Two teachers reported that they could not appraise home practice commitment levels, one had not offered home practice and the remaining seven offered comments that were not directly relevant to the question.

When asked to describe their experiences of participants having their cameras off during online teaching and any impact on teaching and/or sense of the group, 87 participants responded. Most teachers reported that participants’ cameras being off had been a negative experience (*n* = 43). Three sub-themes emerged: impact on group process, teacher discomfort, and concerns for participant safety. Difficulty in engaging with people who were not visible and lack of confidence about participant engagement was reported by 20 teachers. A further 20 referred to their own discomfort around this, primarily because of the impediment they experienced in ‘reading the room’*.* Concern for participants’ safety was implicit in many responses and three teachers specifically cited this as their primary concern when cameras were left off. However, 17 teachers felt that cameras being off had no negative impact on their teaching experience. From the 19 comments that described teachers’ own responses to cameras being switched off, three sub-themes were identified: initiating contact with participants who had left their cameras off, offering guidance regarding visibility, and incorporating camera usage into mindfulness practice in some way.

In response to the question about why participants may have dropped out of the online course (*n* = 71) six themes were identified: time constraints, various changes in circumstances, issues related to being online, pre-existing health challenges, dissatisfaction with the program and no reason provided. The time factor was most frequently occurring (*n* = 29). Within this category, the sub-themes were a general lack of time/not coping with the course (*n* = 16), work commitments (*n* = 11) and family commitments (*n* = 2). A change of circumstances causing drop-out was reported by 17 teachers.

### Teachers’ experiences of the pedagogy of teaching MBPs online

Most teachers found their online teaching experiences comparable with their in-person experiences: just under 70% of teachers found online teaching to be as satisfactory or better than their experience of in-person delivery. Aspects of the online teaching experience were explored in more detail (see Table 5). For those who found aspects of the online teaching less satisfactory than in-person teaching, the findings can be summarised as follows. Reports of somewhat or significantly less participant engagement were made by 25.9% of teachers, and 35.1% stated they felt somewhat or significantly less connected with their group when compared with in-person MBP groups. A total of 25.5% of teachers found it somewhat or significantly less easy to convey course themes online. When asked about their ability to attend to and facilitate group process, 32.1% reported that this was somewhat more challenging and a further 13.2% found this significantly more difficult when teaching online.

**Table 5:** Comparing Online Experience with In-person

**Overall Satisfaction**					
	*N:*	92			
	*n*	%			
Significantly more	8	8.7			
Somewhat more	12	13.0			
Similar	44	47.8			
Somewhat less	25	27.2			
Significantly less	3	3.3			
**Participant Engagement**	*N:*	93			
	*n*	%			
Significantly more	5	5.4			
Somewhat more	10	10.8			
Similar	54	58.1			
Somewhat less	22	23.7			
Significantly less	2	2.2			
**Ability to Convey Themes**	*N:*	106			
	*n*	%			
Significantly more	4	3.8			
Somewhat more	9	8.5			
Similar	66	62.3			
Somewhat less	14	13.2			
Significantly less	13	12.3			
**Connection with Attendees**	*N:*	106			
	*n*	%			
Significantly more	2	1.9			
Somewhat more	5	4.7			
Similar	54	50.9			
Somewhat less	36	34.0			
Significantly less	7	6.6			
Not applicable	12	11.3			
**Ability to Develop Group Process**	*N:*	106			
	*n*	%			
Significantly more	2	1.9			
Somewhat more	9	8.5			
Similar	47	44.3			
Somewhat less	34	32.1			
Significantly less	0	0.0			
Not applicable	14	13.2			
**Extent of Disclosures**	*N:*	92			
	*n*	%			
Significantly more	4	4.3			
Somewhat more	10	10.9			
Similar	48	52.2			
Somewhat less	28	30.4			
Significantly less	2	2.2			
**Ability to Manage Distress**	*N:*	93			
	*n*	%			
Significantly more	0	0.0			
Somewhat more	2	2.2			
Similar	43	46.2			
Somewhat less	43	46.2			
Significantly less	5	5.4			

With respect to participants’ willingness to make significant personal disclosures during class, as might be expected in-person, 32.6% of teachers believed that helpful personal sharing happened somewhat or significantly less frequently online. Almost half—46.6%—of teachers considered that their ability to manage participant distress was somewhat diminished online and a further 5.4% reported that this ability was significantly impaired, in part because such distress would be more difficult to identify remotely. Table 5 compares teachers’ experiences of their most recent online course with their general experiences of in-person teaching.

Having responded to the initial quantitative questions, teachers were invited to comment regarding their experiences of online pedagogy and the content of these responses subsequently analysed for recurring themes. See Table 6 for the themes and sub-themes.

**Table 6:** Content analysis online pedagogy

**Satisfaction Online**		
*N*: 46	*n*	
Online Advantages		
*- Practical*	13	*'Personally, I just appreciate not having to drive and to prepare the venue, this feels very comfortable'*
*- Group Process*	4	*'The classes were smaller and so quite intimate and participants were able to speak a lot and interact well, so good group process happening'*
*- Comfort*	2	*'Because I and the participants didn't have to drive, were in familiar, safe environments, helped to be more at ease'*
*- Other*	1	*'It is certainly easier to teach in person, but I have taught some very lovely groups online and felt very satisfied with the experience'*
Online Disadvantages		
*- Group Process*	16	*'There is a "humane, feeling, compassionate" atmosphere created in live groups which is somewhat missing online'*
*- Outcomes*	3	*'I feel it becomes more 'theoretical' when teaching online and is bound to be less 'embodied' without the physical presence*’
*- Other*	1	*'The quality of online teaching is not as good as offline teaching, but participants' expectation of online teaching is correspondingly low*’
No Strong Preference	3	*'I enjoy both approaches, and am happy to adapt. The group rather than the mode of delivery seem most important'*
Miscellaneous	3	
**Participant Engagement Online**		
*N*: 41	*n*	
Less Engaged Online	15	*'It was easier for people to be distracted in their own environment, they can discount the presence of others'*
Similarly Engaged Online	11	*'Depends on the group: an open and willing group engages with the online course just as well as in in-person courses'*
More Engaged Online	7	*'Surprisingly there is more confidence and easy going mood in the online sessions'*
Couldn't Discern	4	
Miscellaneous	4	
**Teacher Embodiment Online**		
*N*: 131	*n*	
Embodiment Less Online	17	*'Guiding the meditations is different. I miss the presence of others which helps me drop into my body, and yet […]guiding felt extremely intimate.’*
Other Negative Observations	35	*'When in-person, seeing where people are sitting in the circle helps me remember them and what they say / how I feel about them. Online, no "spatial memory"'*
Embodiment Similar Online	44	*'I have found online teaching more "embodied" than I expected and not a great deal different from in person'*
Embodiment Greater Online	13	*'Felt easier in some ways as in isolation of my room and with participants on mute most of the time… less distractions'*
Other Positive Observations	11	*'I felt that it was fine—if anything I felt a little more confident delivering it online as it somehow made me feel like fewer eyes were all on me!'*
Only Taught Online	6	
Miscellaneous	5	
**Ability to Convey Themes Online**		
*N*: 37	*n*	
More Difficult Online	12	‘*Used technology available […] but not as good as being 'in the room' where it feels as though there is more active participation and interaction*.’
Similar Online	8	*’I am also now teaching hybrid classes and find that those in the room and at home have found ways to link and engage in similar ways’*
Easier Online	5	*'Easy to bring in ppt briefly, show text of poems on screen, little video clips, without it disrupting class’*
Miscellaneous	12	
**Connection with Attendees Online**		
*N*: 33	*n*	
Less than In-Person	13	*'Not seeing and sensing the whole body and having eye to eye contact and good sight of faces and eyes limits embodied relational connection’*
Similar to In-Person	11	*'The relationships are developed online as in the room. No difference'*
Depends on the Group	3	*'It depends on the openness of the participants to share and comment, regardless of whether it was an online or in-person group*’
Miscellaneous	6	*'Online participants tend to reach out to me more often between classes than in my in-person classes'*
**Ability to Facilitate Group Process Online**		
*N*: 28	*n*	
Barriers to Group Process	11	*'In-person there is the tangible connection of being in the same room and seeing the whole of a person rather than just a head which I think has a huge effect’*
Feasible Online	6	‘*Group process of course still happens but I think more of it can be hidden, for example, no sense of what is happening in break out rooms*’
What Supported Group Process	6	*'I can see more people at once than in a crowded in-person setting, and the transition back to our seats after movement is much shorter and easier’*
Miscellaneous	5	*'I know it is not recommended, but I have [..]gone round each person asking for input or feedback rather than just inviting feedback […] more group cohesion’*
**Use of Small Groups Online**		
*N*: 51	*n*	
Positive Experience	16	*'Breakout rooms are great for stimulating discussion and building group cohesion*’
Unfeasible on Platform	2	
Negative Experience	1	
Chose Not to Use	1	
Practical Matters		
*- Group Size*	11	*'Breakout groups—made them 3 or 4's mainly. Occasional pairs at last 2 sessions if that felt appropriate for the group'*
*- Allocation*	15	*'I allocated to ensure everyone had a chance over the 9 weeks to work with each other'*
Miscellaneous	4	*'Less participant choice about who they work with in pairs, groups *etc.*’*
**Readiness to Engage with Inquiry Online**		
*N*: 38	*n*	
More Ready	9	*'They are usually at home, which offers much more comfort and security than being elsewhere […]lends itself to rapid, deep, and wide exploration of experience’*
Similarly Ready	7	*'Those willing were just as willing. Had to work harder as facilitator though'*
Less Ready	6	*'Somewhat hesitant to speak—worried about talking over others'*
Techniques to Improve Readiness		
*- Chat Function*	6	*'The option to use chat has I think rely {sic] opened up possibilities for people to engage and the chat can provide options fir {sic} deeper enquiry’*
*- Breakout Room*	2	
*- Questioning*	1	
Miscellaneous	7	*'It feels more light and horizontal as do not wish to go too deep. Harder to monitor people's reactions in terms of a felt sense'*
**Extent of Disclosures Online**		
*N*: 23	*n*	
More Disclosure Online	5	*'I think that participants feeling more relaxed enabled them to open up more.*’
Similar Disclosure Online	5	*'It is similar i.e. very occasional as discouraged in the orientation and teaching*’
Less Disclosure Online	5	*'In the questionnaire, the disclosures were similar, in the online classes, people tend to not disclose or discuss personal issues with me compared to in-person*’
Indeterminate Level	1	
Miscellaneous	7	
**Ability to Manage Distress Online**		
*N*: 54	*n*	
Difficulty Experienced		
*- Identification*	15	*'I think it is harder to assess. I fear I may have missed participants distress but don’t know. As I can’t see them all easily when guiding meditations this is hard*’
*'- Response*	8	*'Difficult to respond in the moment, or immediately after the session'*
No Difficulty Experienced	5	*'One does not have to see the entire body to notice distress. And it’s easier to see fidgety movements in a larger group online*’
Responses Initiated	21	
*- Post-Session*	13	*'In-person, I try to speak with the person before or after class, which is not possible online, so I send an email or call'*
*- Immediate*	8	*'Different…need to address as a group, use body language, potentially use breakout groups'*
Miscellaneous	5	‘*This is a problem area when teaching alone – safeguarding protocols are necessary so that support can be summoned if required’*

When participants were asked to compare their experience of teaching an online and in-person course (*N* = 46), four overarching themes were identified: group process, practicalities, comfort, and outcomes. Some teachers (*n* = 16) noted that delivering MBPs online impeded group process in some way, the absence of in-person interaction and physical presence proving challenging. The benefits of online teaching were cited as practical reasons such as avoiding a commute, not needing to hire a venue, and ease of set up (*n* = 13).

When asked about participants’ engagement with the online taught sessions relative to experiences of a typical in-person course, 41 teachers responded. There were 15 teachers who reported diminished participant engagement online, commenting on multi-tasking, distractions, cameras being turned off, less depth of meditation, perceiving the courses as less deep, or arriving straight from another activity and having less settling time. Others (*n* = 11) felt participant engagement was much the same in both in-person and online courses. A smaller number (*n* = 7) found that engagement was better online, perhaps because participants had not had to travel, or felt more willing to talk. It was noted that the chat function supported engagement and that being online meant that attendance could still happen if circumstances were unfavourable, for example, if the participant was caring for a family member.

The question that generated the most responses (*N* = 131) was about how teachers felt about their ability to embody mindfulness online versus in-person. The themes identified related to level of embodying of mindfulness compared with in-person teaching (*n* = 74) and other observations relating to embodiment online (*n* = 46). Of those who commented, 44 noted similar levels of embodiment when teaching online. Thirteen reported experiencing greater embodiment, whilst 17 felt less embodied online. Challenges relating to being embodied were noted by 32% of teachers (*n* = 35).

The survey asked teachers about satisfaction with their ability to convey course themes online (*N* = 37). There were 12 teachers who stated that it was more difficult to convey course themes online, due to not having physical teaching aids, it being harder for participants to experience the teacher’s embodiment of the practice, and the risk of being too didactic rather than allowing themes to emerge. A further eight experienced comparable ease in conveying course themes compared to an in-person course or stated that some aspects were enhanced in each setting and that they had adapted successfully. Five reported that it was easier to convey course themes online.

Mindfulness-based teachers offered diverse experiences regarding the extent to which they felt connected to course participants when teaching online (*N* = 33). Of those who commented, thirteen wrote that they felt less connected with their participants than had they been in a room together, whilst eleven stated that their sense of connection was comparable with an in-person course. No-one stated that the connection was greater online than in-person.

Teachers were asked about satisfaction with their ability to attend to and facilitate group process—this question generated 28 comments. Three sub-themes were identified: barriers to group process when teaching online (*n* = 11), the feasibility of group process in an online setting (*n* = 6) and techniques used to cultivate group process (*n* = 6), for example breakout rooms and staying online after a session.

Questions concerning experiences of using small groups online (i.e., breakout rooms), including aspects such as group size, how teachers arranged participant allocation and the nature of any technical issues encountered, elicited 51 responses. A positive experience of using breakout rooms was reported by 16 people. Further comments (*n* = 26) related to the practicalities of using breakout rooms. The two sub-themes that were identified here were group size and how to allocate participants to breakout rooms.

When asked about the extent of participants’ readiness to engage with inquiry within online groups, 38 commented, with little concurrence. Some (*n* = 9) stated that their participants showed greater engagement with the inquiry process online, others (*n* = 7) reported that engagement was similar to in-person, or varied from class to class in the usual way, and a further group (*n* = 6) suggested that online students were less ready to engage with inquiry and that it was somewhat easier to stay quiet from behind a screen, or that they were worried about talking over each other. Nine teachers outlined techniques they had used to enhance this readiness to speak: the chat function was mentioned by six: and breakout rooms by two.

Teachers (*n* = 23) also reported on level of participant disclosure online compared with in-person, opinions being evenly split. Seven people provided responses classed as miscellaneous, such as difficulties in knowing whether differences related to the modality, and some stating that MBPs are not the place for such disclosures.

When asked about their ability to respond to signs of participant distress when teaching online, 54 teachers responded. The themes identified included difficulties in identifying and responding to distress, and the types of responses initiated. For 15 teachers it had been difficult to identify distress online. There were 13 who followed up the participant’s distress after the session by making contact specifically to check on their wellbeing. A further eight mentioned that they addressed distress during the session. The means by which they did this included incorporating the distress into inquiry, i.e. openly exploring the participant’s experience within the group as a means of modelling mindful approaches to distress. This is comparable with what might happen in person. An alternative approach taken was to meet the distressed participant one-to-one, in a breakout room.

## Discussion

Teachers’ responses suggest that their experience of delivering MBPs online was generally a positive one, which is comparable with their experiences of in-person training, albeit with some key differences and considerations. Processes such as participant engagement, drop-out rates, and commitment to home practice were considered similar to those encountered in-person, and experiences of technology challenges (either for teacher or participant) were not reported by the overwhelming majority. The notable differences between teacher experiences of online and in-person delivery concerned group process, teacher embodying of mindfulness, and safeguarding. The three areas we initially identified for consideration (who teaches mindfulness online, which MBP curricula are taught online, to whom, and what teachers think of teaching mindfulness online) will be explored in turn.

### Who teaches mindfulness online?

The study identified that, of 109 teachers that responded to the survey, the majority were white, middle-class, middle-aged cis-gender females, who were highly educated, and lived in households more affluent than the UK average. Teacher demographics are of increasing interest in the field, with observers noting that MBPs have largely been delivered to and by those who are privileged by the social hierarchy [23]. It has been suggested that the potential of mindfulness programs to reach broader sectors of society is somewhat negated by the narrow demographics from which those engaging in mindfulness have been drawn [31]. It is difficult to say how representative this sample of 109 MBP teachers is of the wider field, as there is no published data which has attempted to identify the number of practicing MBP teachers worldwide, nor their demographics. It does, however, align with observations of the field, where it is acknowledged that there is work to be done to adequately include marginalised voices [23].

If barriers to accessing mindfulness are reducing due to increased online delivery and yet MBPs are taught by a narrow section of society, there are likely to be obstacles to participation that may remain invisible to those offering the trainings. The identity characteristics of the people who teach MBPs may significantly influence participants’ ability and willingness to join trainings (whether online or in-person), due to issues such as participants’ perception of whether they will feel comfortable in a specific teacher’s group. Others have found that trainee MBP teachers who came from minority groups felt more included in the group if they felt represented and acknowledged by their trainers [32]. They found that feelings of safety impacted the extent to which trainee MBP teachers felt able to make personal disclosures. Without a sense of safety, the potential for learning is likely to be diminished.

Many in the field have been calling for increased diversity among MBP teacher trainers for some time, and also acknowledge that there is much to be done to decolonise MBP teaching [22]. There has been a call to ensure that all MBP teachers pro-actively engage in anti-oppression awareness building, and this seems essential if mindfulness teaching is to connect with diverse participants’ lived experiences.

Engaging in MBP teacher training is costly and has historically been available via a small number of organisations offering in-person programs. It may be possible that financial barriers to accessing MBP training have contributed to a lack of diversity in the MBP teaching population. An important aspect of MBP learning involves the participant allowing themselves to be vulnerable; conditions are far more likely to be conducive if they are not made to feel different from the teacher and wider group. Financial obstacles appear to be the primary barrier to mindfulness teacher training [32], and it seems likely that interplay between financial means and other demographics will strongly influence who is able to access MBP teacher training. Teacher training bursaries favouring those from marginalised groups do exist but could be further prioritised, particularly given the enhanced access to teacher training afforded by online delivery. It seems that the field needs to attend to the interface between equity, diversity, and inclusion and access to teacher training, the design of curricula and their pedagogies, and ongoing good practice requirements.

Research suggests that MBP participation can foster prosocial behaviour and support skilful engagement with some of the most significant challenges facing the world in the twenty-first century, for example racial injustice and the climate crisis [23]. Collaboration between diverse individuals and societies is going to be essential if structural inequality and the social, racial, and economic injustices which also contribute to major public health challenges are to be addressed.

### Which MBP curriculums are taught online, to whom?

The MBP curriculums that are being taught online appear comparable with in-person offerings (i.e., both established and more niche curricula), and were typically offered over a similar time frame and duration as in-person courses (for example in-person Mindfulness-Based Stress Reduction [MBSR] sessions last for 2.5 h, and online MBSR teachers usually reported that their sessions were of the same duration). A significant proportion of teachers reported not having charged for online MBP courses. We do not know the extent to which this was because they were working in workplace and healthcare settings where the program was offered free of charge to the participant, or if this reflected a shift towards MBPs being offered free of charge online during the global pandemic. Further research is needed to investigate the impact of free mindfulness programs for those whose livelihood is dependent upon mindfulness teaching. Whilst free courses eliminate financial barriers to participation in MBPs, this trend reduces the potential for engagement from teachers relying on paid work to make a living.

### What teachers think about teaching mindfulness online

Many MBP teachers were pleasantly surprised by the efficacy of the online medium and commented on the practical benefits of working online. Teachers welcomed the greater diversity in their online groups when compared to in-person (pre-pandemic). Online groups tended to cover a wider age range when compared with the typical in-person group which would anecdotally have consisted of primarily middle-aged and some older adults (i.e., over 65 years old). Within this extended age range, some teachers reported fewer older adults than they would expect to teach face-to-face, and substantially more young people than would be usual for in-person courses. This finding has also been replicated in research into online therapy, which suggested that online platforms are more comfortable and suitable for children, adolescents, and young adults [33]. Some teachers noted more male participants than expected, and geographical diversity was almost universally increased. Online participants were able to overcome barriers such as health issues or caring responsibilities that would typically present an insurmountable obstacle to attending an in-person program. These findings are important as diversity influences group formation and learning experiences and can highlight both the differences in and commonality of human experience, important implicit themes in MBPs.

A significant number of MBP teachers thought that online teaching could impede group process, potentially affecting learning outcomes [19]. Teachers reported feeling less connected with their participants, which has implications for the co-creation of the group space [20]. An exploration of veteran military personnel’s experiences of in-person and virtual world MBP training identified a correlation between trust, class satisfaction, and didactic learning [34]. These results showed that participants reported greater trust when they had experienced in-person courses compared with virtual world learning, suggesting that proactive pedagogical strategies for building trust between the teacher and participants should be a particular priority for online learning contexts.

### Pedagogical issues of online teaching

Many teachers commented on differences in their perception of their own capacities to embody mindfulness online. As described in MBI:TAC [25], embodying is the vehicle through which the practice of mindfulness is tangibly sensed in the learning space. Embodying allows the mindfulness teacher to convey the essence of mindfulness implicitly, through their non-verbal presence and how they hold themselves within the teaching process. Since learning mindfulness is an experiential, relational process and many important elements of the teachings are delivered implicitly, participants might ‘catch’ mindfulness if embodying is evident in the teaching space [25]. Even when satisfied with their experience of embodying, most teachers, despite being experienced in mindfulness teaching, perceived challenges in communicating their embodying online because the whole of their body was not visible to other group members in the way it would be when teaching in-person. This is of concern given that embodying is considered a critical factor in enabling MBP participants to move towards the ‘felt sense’ or experiential understanding of the attitudes of mindfulness [35]. Embodiment of mindfulness was found to be the most difficult mindfulness domain to rate reliably using an audio-only recording format [36] supporting the original findings of Crane et al. who, when establishing the MBI:TAC, suggested that embodiment was the most challenging domain to articulate [37]. It seems important to consider how communication of embodying may be enhanced online, perhaps through techniques such as spotlighting the teacher, so that participant focus is directed exclusively towards the teacher rather than to other group members. It is possible that technological advances may permit participants to experience different views of the teacher and if so, the functionality seems worth exploring in the context of supporting the teacher in conveying embodying via video conferencing.

Several teachers commented that they felt more readily embodied when teaching online as they experienced fewer interruptions from external distractions than would be typically expected at an in-person venue, which improved their sense of presence and alertness. Nevertheless, it seems that effectively conveying the attitudinal foundations of mindfulness whilst enabling remote participation and cultivating group process may warrant more nuanced training and assessment than has hitherto been offered, particularly as the online modality is now mainstream.

The study’s findings suggest that online groups are likely to be more diverse than those found at in-person courses, where attendees can reasonably be expected to at least have geography in common. It seems possible that adjusting MBP curricula to place greater emphasis on group formation, before, during and after sessions may be of significant benefit to teachers and participants alike. Techniques might include more social opportunities such as breaks and specific group formation tasks, to support a feeling of community between individuals who are unlikely to ever meet in person.

Regarding conveying course themes, some teachers reported a perception that online teaching can be more theoretical and didactic and somewhat less invitational than in-person courses. Several teachers described feeling that it was more difficult to patiently await participant contribution than it would be in-person, leading to the teacher requesting responses from all participants via a ‘go round’, or through use of the chat function. They also reported that the use of the online chat function encouraged lighter touch engagement. These altered relationships with invitation and inquiry led some teachers to perceive that the approaches they adopted to suit the modality did not feel fully aligned with their training; adjustments were typically described as a pragmatic response to the challenges of delivering mindfulness via a screen. Identifying subtle shifts in the relational aspects of program delivery seem important given that the invitational nature of mindfulness courses has been identified as an essential ‘warp’ (i.e., non-negotiable) element of all MBPs [1] and effective inquiry is crucial to the mindfulness learning journey. It seems important to explore the impact of the online medium on these subtle aspects of experience and to support teachers in cultivating the complex skills needed to retain the warp elements of MBPs, irrespective of delivery mode.

Teachers shared concerns about participants having their cameras turned off and the impact of this on group process and participant safety. Explicit guidance is available in the MBI:TAC addendum focused on the pedagogical specifics of online MBP teaching [11]. This states that teachers should ideally be able to always see all the participants when teaching online, yet it is clear from survey responses that a variety of approaches to participant visibility are being adopted and that seeing everyone is not always practically possible. Teachers can request that cameras are turned on, but ultimately that decision is in the control of their participants, and to insist on cameras on contravenes the invitational nature of the program, and so remains a debate in the field with more research needed about how different approaches may impact participant engagement. A recent study on students’ perceptions of video use during synchronous distance learning [38] showed that students viewed cameras being on as crucial in promoting discipline, self -control, community and assisted with cooperation among students. It may be that the MBP field needs to further develop guidance regarding camera use in specific scenarios and contexts to optimise teachers’ ability to safeguard their participants. Camera usage also seems important with respect to building trust and safety and boundaries that will support group process [19] – it seems likely that specific training about developing the group online, and particularly with regard to adapting delivery of curricula to include specific opportunities for cultivating greater ‘presence’ would be supportive of both teachers and participants.

Concerns were raised regarding the ability to identify and manage participant distress in an online setting. It seems important for teachers to plan for these tender moments, establishing expectations regarding online etiquette. There is clear guidance around how best to approach these and other areas of concern, such as the increased risk of privacy invasion online, in the MBI:TAC addendum [11]. Nevertheless, based on the responses received regarding managing participant distress, this appears to be an area where teachers might welcome more explicit guidance.

### Pedagogical implications

It seems likely that online delivery presents some challenges to quality of teaching in the field and to teacher training. For instance, when teaching online, it might be feasible for a teacher to use notes to support their teaching in a way that would undermine their authenticity in-person. People teaching mindfulness online may therefore manage to convey a sense of being more skilled than they are, which could impact participant experience and safety. This highlights the importance of teachers in the field being able to demonstrate reputable training experiences and ongoing adherence to Good Practice Guidelines [4]. Online delivery may render MBI:TAC-based competency assessment more difficult for supervisors and for those involved in online teacher training, requiring particularly expert assessment compared with when a trainer or assessor is appraising embodiment in-person. The remoteness of the teacher from participants appears to increase the risk of safeguarding concerns and so explicit guidance about how to protect participants, from orientation through to best practice for handling a challenging situation remotely, could be beneficial. Similarly, this remoteness creates challenges around engagement; it is possible that the field should look at how to convey expectations and create a supportive container that fosters deeper engagement. It is possible that curricula may need adapting to offer shorter sessions than are conventional in-person, to reflect participant (and teacher) challenges when spending lengthy periods online. The online medium appears to offer interesting opportunities for supporting longer term practice, (e.g., through informal drop-in or follow up sessions) so perhaps a shift towards shorter but more frequent doses of mindfulness training may better support participant learning, and further research is needed. Although mindfulness principles remain consistent across teaching modalities, it seems important that supervisors overseeing online teaching have had first-hand experience of the online medium. Highlighting the subtle differences that might be encountered between the modalities could be addressed during supervision training.

Many of the challenges experienced by mindfulness teachers and trainers when working remotely are likely to be encountered in other online group learning environments, where research suggests that the teachers must be familiar with internet-based technologies, adjust their teaching plans and methods, strengthen their interactions and sustain student engagement [39].

### Future research

Future research could further explore pedagogical aspects of online mindfulness teaching such as embodiment and group process. Concerning embodiment, for example, it could be helpful to further explore whether it was teachers experience of embodying that differed online, and/or their ability to convey that embodiment to their participants. Fine-tuning questions around group process and pedagogy would also build on the findings of this study.

Given that it seems that online delivery of MBPs is here to stay, and some teachers have experienced a sense of loss when teaching via that modality, it seems important for future research to explore what would enhance the experience for participants and what they need from training organisations and supervisors to facilitate development. The losses, although not universally experienced, were most keenly felt around relational aspects of the teaching, including fewer opportunities to connect, a different quality of connection and challenges regarding group process. Other issues included perceived diminished depth of learning; less engagement from participants; more scope for distraction or interruptions when online at home, and concerns around safeguarding and handling difficult situations remotely. It is possible that MBP curricula and session structures would benefit from in-depth review to ensure that the ‘warp’ elements [1] are optimised, despite the shift in delivery mode. Research into the MBP session length and frequency that would offer the optimal experience in the online setting would be of interest. Gaining more insight into teachers’ views of how to blend the strengths of online training whilst ensuring that the implicit curriculum is retained and developed would also be a valuable research theme.

There is scope for additional research into the demographics of those who teach mindfulness and how this may impact those who attend MBPs. Additionally, there is no literature available regarding who researches mindfulness, and better understanding the demographics of researchers also seems important.

### Limitations

It is likely that at the time of responding (which was during what was, for many, a lockdown period, or soon after) most teachers had taught their most recent MBP as an online course. It may be that when asked to compare this most recent online course with their experiences of teaching in-person, that positive or negative experiences of working online may have been exaggerated, or similar experiences encountered in-person may have been overlooked. Using self-report meant that we were reliant on teachers’ awareness of various subjective and relative components such as their own skills, participant engagement, diversity, and so on. There are inevitably shortcomings associated with using self-report data. Differences in perspectives may be influenced by varying levels of experience with online teaching (some teachers had only taught one course online whereas others had taught over twenty) and whether teachers were forced into teaching online due to COVID-19 restrictions or had intentionally opted into the modality. It is possible that responses may differ if the study were repeated now that online teaching has become more familiar to much of the world’s population. It also seems likely that perspectives will have been influenced by prior teaching experiences. Whilst survey respondents were typically experienced teachers, there was a significant range of experience (from delivery of one course to more than 140) in the last five years. Although teacher development is non-linear, it is probable that teaching experience will have influenced responses to questions regarding pedagogy. The factors that influence teachers’ experiences when delivering MBPs online would be an interesting topic for future research.

The comparison of fees charged by individuals was limited because we asked people what they charged without asking for contextual information and were unable to distinguish between those who had chosen not to charge and those working in settings where MBPs are offered free of charge. Without this data it is not possible to draw any inferences from these findings.

This study invited participation from international mindfulness teachers. However, it is difficult to assess the representativeness of responses received, as there is no data on the number of MBP teachers trained or currently working, nationally or worldwide. Furthermore, the snowballing approach to promoting the survey may have introduced bias as it may have been snowballed to ‘similar others’ and is not guaranteed to have reached diverse MBP teachers and may over-represent the organisations contacted first [40]. MBPs are not equally accessible around the world for a variety of reasons, including that MBP training organisations have historically been European and American. This is a rapidly changing landscape and the opportunities for international training (for both teacher and participant) have been significantly expanded by the enhanced accessibility of mindfulness teacher training online. The demographic data must therefore be interpreted with a degree of caution, given that we were unable to demonstrate the extent to which it represents the international mindfulness teaching population. Future studies should therefore aim to capture mindfulness teachers’ experiences across the broadest possible range of nationalities and cultures.

## Conclusions

Overall, this experienced group of teachers found teaching MBPs online generally satisfactory, but limitations and challenges were experienced, particularly regarding embodying, relational connection with participants, group process, and managing participant safety. Teachers, overall, represented a narrow demographic and welcomed the geographic diversity of their participants as well as the many practical benefits of the online modality. Although online delivery of MBPs was initially a practical response to the restrictions on movement imposed because of the pandemic, it is now an established format. This shift to online teaching has implications for MBP teacher training, recruitment, assessment, curricula, good practice guidelines, and supervision, as well as for MBP delivery. The study’s findings suggest that the mindfulness field – teachers, trainers, researchers, and those shaping the field – would benefit from making explicit the subtle differences experienced by teachers when working online. This might include developing teacher training curricula, assessment guidelines and best practice advice to focus on the more challenging aspects of online teaching, particularly around embodying, group process and participant safety. Addressing these challenges, particularly in highly nuanced pedagogical areas, should be a high priority for the mindfulness field since the existing prevalence and potential reach of online delivery has potential to significantly influence transformation, at both individual and societal levels.

## Data Availability

The original dataset analysed during the study is not publicly available, to protect participant privacy.

## Abbreviations

MBP: Mindfulness-Based Program
UK: United Kingdom
NICE: National Institute of Clinical Excellence
MBCT: Mindfulness-Based Cognitive Therapy
BAMBA: British Association of Mindfulness-Based Approaches
GBP: Great British Pounds
MBI:TAC: Mindfulness-Based Interventions: Teaching Assessment Criteria
SPSS: Statistical Package for the Social Sciences
MBSR: Mindfulness-Based Stress Reduction
USA: United States of America
